# Lovastatin as a supplement to mitigate rumen methanogenesis: an overview

**DOI:** 10.1186/s40104-021-00641-8

**Published:** 2021-12-16

**Authors:** Amaury Ábrego-Gacía, Héctor M. Poggi-Varaldo, Vania Robles-González, Teresa Ponce-Noyola, Graciano Calva-Calva, Elvira Ríos-Leal, Daniel Estrada-Bárcenas, Alfredo Mendoza-Vargas

**Affiliations:** 1 Department of Biotechnology and Bioengineering, CINVESTAV-IPN, P.O.Box 17-740, 07000 Mexico City, Mexico; 2 Environmental Biotechnology and Renewable Energies Group, CINVESTAV-IPN, P.O.Box 17-740, 07000 Mexico City, Mexico; 3 Instituto de Hidrología, Universidad Tecnológica de la Mixteca, Oaxaca 69000 Huajuapan de León, Mexico; 4 National Collection of Microbial and Cellular Cultures, CINVESTAV-IPN, P.O.Box17-740, 07000 Mexico City, Mexico; 5 Unidad de Secuenciación e Identificación de Polimorfismos, Instituto Nacional de Medicina Genómica, 14610 Mexico City, Mexico

**Keywords:** Fermentation, Lovastatin, Methanogenesis, Microbiota, Rumen

## Abstract

Methane from enteric fermentation is the gas with the greatest environmental impact emitted by ruminants. Lovastatin (Lv) addition to feedstocks could be a strategy to mitigate rumen methane emissions via decreasing the population of methanogenic archaea (MA). Thus, this paper provides the first overview of the effects of Lv supplementation, focusing on the inhibition of methane production, rumen microbiota, and ruminal fermentation. Results indicated that Lv treatment had a strong anti-methanogenic effect on pure strains of MA. However, there are uncertainties from *in vitro* rumen fermentation trials with complex substrates and rumen inoculum.

Solid-state fermentation (SSF) has emerged as a cost-effective option to produce Lv. In this way, SSF of agricultural residues as an Lv-carrier supplement in sheep and goats demonstrated a consistent decrease in ruminal methane emissions. The experimental evidence for *in vitro* conditions showed that Lv did not affect the volatile fatty acids (VFA). However, *in vivo* experiments demonstrated that the production of VFA was decreased. Lv did not negatively affect the digestibility of dry matter during *in vitro* and *in vivo* methods, and there is even evidence that it can induce an increase in digestibility. Regarding the rumen microbiota, populations of MA were reduced, and no differences were detected in alpha and beta diversity associated with Lv treatment. However, some changes in the relative abundance of the microbiota were induced. Further studies are recommended on: (*i*) Lv biodegradation products and stability, as well as its adsorption onto the solid matter in the rumen, to gain more insight on the “available” or effective Lv concentration; and (*ii*) to determine whether the effect of Lv on ruminal fermentation also depends on the feed composition and different ruminants.

## Introduction

Recently, the contribution of the ruminant livestock sector to greenhouse gas (GHG) emissions has become of concern and increasingly crucial for animal and environmental scientists. The main GHGs of this sector are methane (CH_4_) and nitrous oxide (N_2_O) [1, 2]. Methane emitted by livestock from enteric fermentation is the gas with the greatest environmental impact. From a climate change point of view**,** CH_4_ has been reported to be the most abundant GHG other than CO_2_ [3]. The magnitude estimation of CH_4_ compared to CO_2_ differs based on its higher global warming potential (28 times over a 100-year-time horizon) and shorter atmospheric lifetime, which is about 12.4 years [4]. Global GHG emissions from the livestock sector from 1995 to 2005 were between 5.6 and 7.5 gigatons of CO_2_ eq./year and represented ~ 14.5% of the global anthropogenic GHG emissions [2, 5]. Furthermore, worldwide enteric methane emissions were estimated to be approximately 111 Tg/year and contributed almost one-third of global anthropogenic emissions [4].

The main end-products of ruminal fermentation besides methane are volatile fatty acids (VFA) and microbial protein. These products are absorbed in the digestive system and incorporated into the metabolism of the animal host [6]. However, some fermentation end-products (CO_2_ and H_2_) are not absorbed in the rumen. In this case, CO_2_ and H_2_ are consumed by methanogenic archaea (MA) to produce methane, which the animals release into the atmosphere [7]. Also, it is considered that around 2% to 12% of the total energy consumed by ruminants can be metabolized to CH_4_ [8].

Researchers have investigated various approaches to mitigate ruminal methane emissions and enhance livestock productivity aiming for sustainable development [9, 10]. In this context, Lovastatin (Lv) is a competitive inhibitor of the HMG-CoA reductase enzyme (3-Hydroxy-3-methylglutaryl CoA reductase) [11]. The latter is the rate-limiting enzyme for the mevalonate pathway required for the biosynthesis of polyprenols, which have a significant role in maintaining the function and structure of the MA membrane [12].

Therefore, this paper provides the first overview of the effects of Lv supplementation, focusing on the inhibition of methane production, rumen microbiota, and ruminal fermentation.

The manuscript critically examines the role of Lv in the following topics: (*i*) inhibition of MA, (*ii*) *in vitro* and *in vivo* ruminal methane mitigation trials, (*iii*) the pattern of change in rumen fermentation and digestibility, and (*iv*) shifts in the rumen microbiota.

## Lovastatin as a strategy to inhibit methanogenic archaea

Several strategies to mitigate rumen methane emissions have been developed, such as nutrition (lipid supplementation, concentrate based diets), chemical inhibitors (3-nitrooxypropanol, 10-anthraquinone, nitroethane), secondary plant compounds (tannins, flavonoids, saponins), and Lv addition to the feedstocks of ruminants, among others. One crucial issue that could dictate the adoption and success of such strategies is their feasibility of application at the farm level [9, 10, 13].

Furthermore, the adaptive capabilities of livestock production systems must prioritize profitability and food safety to adopt a methane reduction strategy [5]. In this regard, modulation of the rumen is an option for inhibiting methanogens [14].

**Fig. 1:**
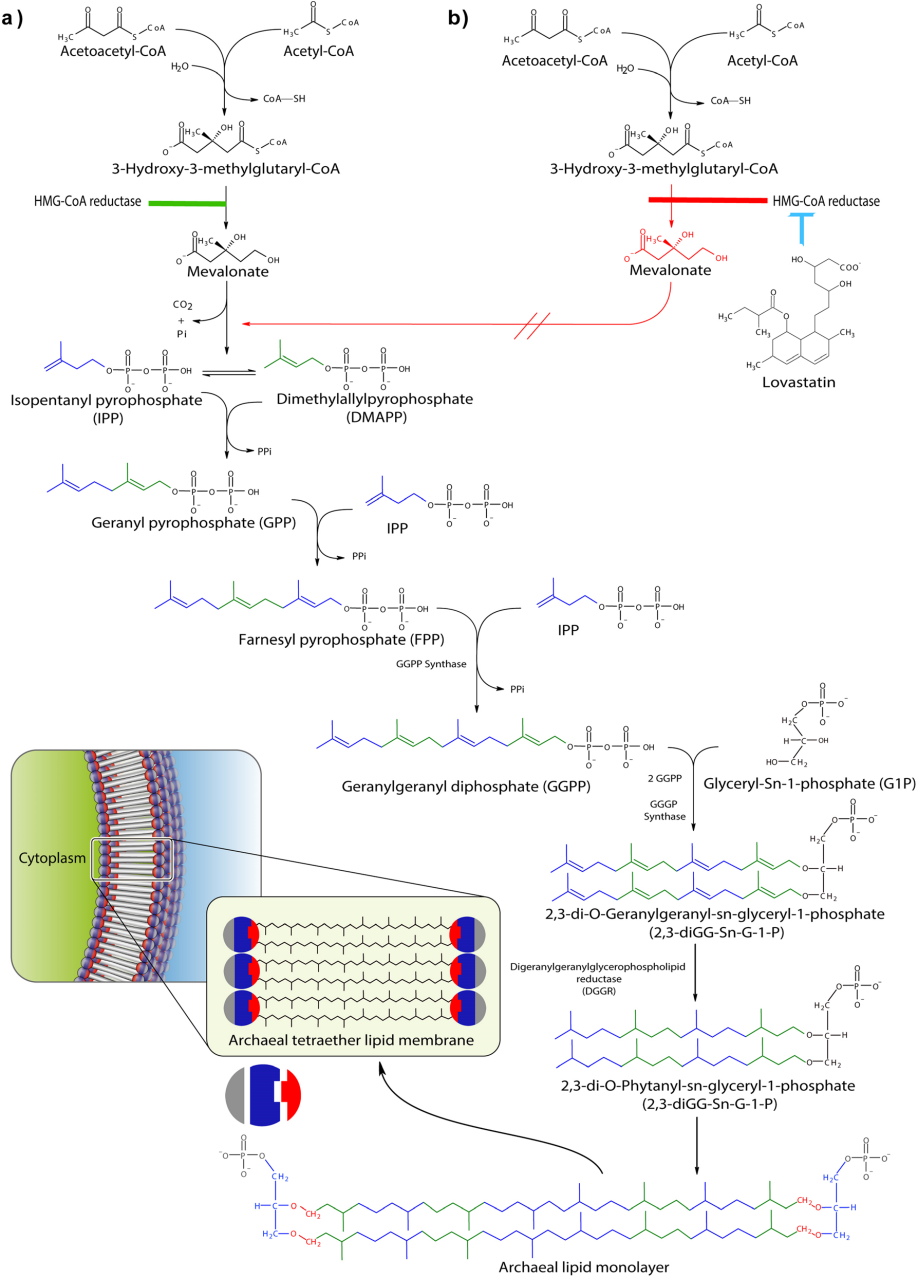
Biosynthesis of archaea membrane ether lipids. **a** Mevalonate pathway and archaeal lipid synthesis. **b** Inhibitory effect of the lovastatin on HMG-CoA reductase, the rate-limiting enzyme in the conversion of HMG-CoA to Mevalonate [12, 17]

It is known that the membranes of MA and eubacteria generally consist of a double-layer or a monolayer of lipid molecules where proteins can float [15]. Unlike eubacteria and eukaryotes, the lipid composition of the archaea consists of chains of isoprenoids linked to the sn-glycerol-1-phosphate backbone by ether bonds [16]. It has been reported that the fundamental unit of archaea membrane lipids could undergo an intermolecular dimerization building a diglycerol-linked pair of C40 isoprenoid hydrocarbon. The archaea membrane synthesizes complex isoprenoid ether lipids (Fig. 1a), which are unique features of the domain archaea [18]. As a result, a strategy to inhibit the growth of MA is via compounds that inhibit the activities of some key enzymes linked to the synthesis of isoprene units.

Statins are MG-CoA reductase inhibitors that catalyze the conversion of HMG-CoA to mevalonic acid (Fig. 1b) [19, 20]. The latter is the pathway for synthesizing isopentenyl pyrophosphate and its isomer dimethylallyl pyrophosphate, important precursors of molecules such as isoprenoids, cholesterol, and terpenes [17, 21].

Statins are categorized into three categories: (*i*) natural statins, which are produced mainly by fungal fermentation, for instance, Lv and pravastatin, (*ii*) semi-synthetic statins that are derived from a natural statin by chemical synthesis here include simvastatin, and (*iii*) synthetic statins which cannot be naturally produced or by chemical synthesis of natural statins, e.g., rosuvastatin, pitavastatin, atorvastatin, fluvastatin, and cerivastatin. Those statins contain two units, i.e., a chiral 3,5-syn-diol acid and a chiral β-hydroxy-γ-lactone (alternatively its open-chain analog) [20, 22].

Among the statins, Lv has attracted great interest as an anti-methanogenic compound. It is a non-hygroscopic crystalline powder, has the empirical formula C_24_H_36_O_5_, a molecular weight of 404.5 g/mol, and its water solubility is 0.4 mg/L [19]. It is soluble in N-dimethylformamide and acetone; it is highly soluble in CHCl_3_. Lv exhibits moderate solubilities in methanol, ethanol, acetonitrile, and isopropanol [23].

The research to date has demonstrated that Lv inhibits MA by two pathways. The first is related to the cell membrane; MA contain long chains of isoprenoid ether lipids as the main components of the cell membrane, which are synthesized via the mevalonate pathway where HMG-CoA reductase acts as the rate-limiting enzyme [24]. Lv inhibits HMG-CoA reductase, thus disrupting the cell membrane synthesis of MA and impeding the membrane-bound electron transport of the pathway for methane production [25]. Finally, the growth of methanogens was negatively affected [26, 27].

The second effect of Lv on inhibition of MA is associated with the F_420_ coenzyme. Several oxidation/reduction reactions are involved in the metabolism of methanogens, which require different oxidoreductase enzymes, some of which participate in the electron transfer during the methanogenesis pathway. For instance, Sharma et al. [28] reported in a prediction model for NADP oxidoreductase inhibition in the *Methanobrevibacter smithii* strain that Lv and mevastatin showed a higher affinity for this enzyme than the F_420_ coenzyme. So, statins could inhibit the activity of the NADP oxidoreductase protein dependent on the F_420_ coenzyme, an electron carrier in the methanogenesis pathway. However, the experimental evidence on this issue is still unclear, and the number of studies considered is small [28, 29].

## Effect of lovastatin on ruminal methane production

Lv is widely indicated for the treatment of hypercholesterolemia in humans [11]. The cost of Lv is approximately 7.5 US $/g of industrial-grade ([30], Sigma-Aldrich, St. Louis MO, USA). Consequently, its use in animal feeding is restricted [26], and some cost-effective alternatives to produce Lv have been evaluated. For instance, Lv production by solid-state fermentation (SSF) has emerged as a suitable option [31].

The SSF and the fermented substrates applied to mitigate rumen methanogenesis have the following advantages [32, 33]: (*i*) lignocellulosic agricultural residues used for SSF are inexpensive, readily available in farms, and commonly utilized for livestock feeding, (*ii*) the fermented substrates should be utilized without pretreatment reducing polluting discharges to the environment from the Lv extraction process, and (*iii*) it can give high-quality yields of secondary metabolites and cellulolytic enzymes.

Several agricultural residues have been assessed as a substrate to produce Lv by SSF, but in this review, only the results of SSF trials where fermented agricultural residues were used to inhibit the methanogenesis are discussed. Interestingly, all strains tested belonged to *Aspergillus terreus*. The SSF of rice straw gave Lv yields of 0.26 and 0.69 mg/g DM [34, 35]. Despite the high-fiber content of rice straw, the Lv productivity was close to that of rice grain (0.57 mg/g DM) [36]. Another substrate in SSF for this target was palm kernel cake (PKC) which resulted in the maximum production of 0.85 mg/g DM [37], and finally, the oat straw, which was the highest Lv yield (23.8 mg/g DM) [31]. These results generally suggest low to moderate Lv yields compared to the findings of experiments devoted to statins production [30].

## *In vitro* experiments

Table 1 summarizes the *in vitro* rumen fermentation data associated with methane mitigation by pure Lv or fermented residues as an Lv-carrier treatment.

**Table 1:** Effect of lovastatin on *in vitro* ruminal methane production

Source of Lv	Donor animals and experimental diet	*In vitro* gas production technique	Lv, mg/L	Methane Inhibition, %	Reference
**Pure cultures of methanogenic archaea**					
Sigma-Aldrich, St Louis, MO, USA		Technique: Hungate tubes Culture medium: BRN Inoculum: *Methanobrevibacter* strains	4	99	[26]
Fermented rice straw extracts		Technique: serum bottles Culture medium: Balch 1 (DSMZ, Germany) Inoculum: *M. smitthii*	50	100	[27]
Sigma-Aldrich, St Louis, MO, USA		Technique: Hungate tubes Culture medium: SAB Inoculum: *M. smitthii*	4	100	[38]
**Ruminal-based inoculum and diets**					
Sigma-Aldrich, St Louis, MO, USA	Ruminant: bovine Diet: f:c^a^ ratio of (50:50)	Technique: fermentation bottles Substrate: diet with a f:c ratio of (50:50) Inoculum: ruminal fluid	5	NS^b^	[39]
Sigma-Aldrich, GmbH, Buchs, Switzerland	Ruminant: bovine Diet: hay, ryegrass, and concentrate	Technique: RUSITEC system Substrate: diet with a f:c ratio of (50:50) Inoculum: ruminal fluid	150	40	[40]
Fermented rice straw	Ruminant: bovine Diet: f:c ratio of (40:60)	Technique: calibrated glass syringes Substrate: fermented rice straw Inoculum: ruminal fluid	4.3	24	[34]
Sigma-Aldrich, St Louis, MO, USA	Ruminant: bovine Diet: f:c ratio of (60:40)	Technique: serum bottles Substrate: diet with a f:c ratio of (50:50) Inoculum: ruminal fluid	3.2	NS	[41]
Fermented rice	Ruminant: sheep Diet: hay	Technique: Hungate tubes Substrate: fermented rice Inoculum: ruminal fluid	40	9.6	[36]
Fermented purple corn stover	Ruminant: dairy steers Diet: no reported	Technique: serum bottles Substrate: fermented purple corn cob Inoculum: ruminal fluid	29.5	14.6	[42]
Fermented Oat straw	Ruminant: bovine Diet: f:c ratio of (60:40)	Technique: serum bottles Substrate: diet with a f:c ratio of (30:70) Inoculum: ruminal fluid	150	38	[31]
Simvastatin, Sigma-Aldrich, Prague, Czech Republic	Ruminant: bovine Diet: f:c ratio of (70:30)	Technique: serum bottles Substrate: diet with a f:c ratio of (64:36) Inoculum: ruminal fluid	100	26.2	[43]

^a^Forage: concentrate ratio

^b^Non-significant

The addition of Lv provides reliable evidence of complete inhibition of methane formation by cultured methanogens. Miller and Wolin [26] evaluated the addition of pure Lv (4 mg/mL) on strains of the *Methanobrevibacter* genus co-cultured with fibrinolytic/cellulolytic bacteria. This dose decreased the growth of the *Methanobrevibacter* as well as CH_4_ production. These results were confirmed by Demonfort et al. [38], who reported for pure cultures of MA and bacteria from the human digestive system that methanogenesis was inhibited entirely at the same concentration of Lv (4 mg/mL). This view also was supported by Jahromi et al. [27], who found that methane production of *Methanobrevibacter smithii* strain was inhibited by 96% with an Lv dose of 50 mg/mL, under similar cultivation conditions to those in the previous research.

Unfortunately, there is no general agreement about the effects of pure Lv on *in vitro* trials using rumen fluid inoculum (RFI) and forages as well as total mixed rations. First, Busquet et al. [39] evaluated a diet with forage to concentrate (f:c) ratio of 50:50, RFI from a dairy cow, and a dose of Lv of 5 mg/L. There was no significant reduction of methane production between treatment and control groups. These results agreed with those of other studies that reported no effects of Lv (at dose 3.2 mg/L) on methanogenesis with grass silage and barley grain ration of (50:50) as substrate and RFI from steers [41]. A possible explanation for these results may be the low Lv dosage; it was initially proposed that methane inhibition was highly significant at 4 mg/L [26] but, this dose was based on pure cultures of MA without substrate and RFI. However, the addition of simvastatin (purity > 97%) at a low dose of 10 mg/L reduced the *in vitro* rumen methanogenesis only by a poor 9.3% using a high forage diet (*P* < 0.05) [43]. The latter is the only article research that reported the anti-methanogenic effect of a low-dosage statin with RFI and a diet to the best of our knowledge.

The role of the presence of solids in the ruminal fermentation tests as well as the complex composition of dissolved and colloidal organic matter could affect the Lv availability for inhibiting the MA possibly due to the well-known phenomenon of hysteresis that characterizes the interaction of soluble organic compounds and organic particles in soils and sediments in the field of remediation [44, 45]. For instance, there is no information on the possible extent of adsorption of Lv onto the solids (e.g., from diets) present in the complex medium (and the opposite process of desorption). If adsorption of Lv were significant, then the Lv concentration available for inhibiting the MA would be a small fraction of the added dosage of Lv [46, 47]. That is, there would be a “sequestration” of Lv that undoubtedly would decrease its inhibitory effect.

In contrast to earlier findings, Soliva et al. [40] observed a 40% inhibition of methane production in an experiment using the “rumen simulation technique.” They used a substrate that consisted of a diet based on barley, ryegrass hay, and soybean, RFI from a dairy cow, and a pure Lv dose of 150 mg/L. The inhibition level observed was consistent with the methane mitigation by pure simvastatin 100 mg/L (*P* < 0.05) incubated with a 70% forage diet and RFI [43].

On the other hand, the experiments with agricultural residues as Lv-carriers showed the following results. Jahromi et al. [34] reported a 24% inhibition of CH_4_ formation with fermented rice straw (concentration of Lv, 4.32 mg/L RFI medium). A later study claimed that fermented purple corn stover (Lv dosage was 29.5 mg/L RFI medium) had a minor effect on decreasing methane production by 14.6% [42]. As noted above, in both cases, the fermented agricultural residues as Lv-carriers were used as the only substrate for *in vitro* rumen fermentation trials.

Recent evidence suggested that replacement of ordinary oat straw (18.9% and 28.4%) with fermented oat straw as an Lv-carrier to achieve initial doses of 100 and 150 mg Lv/L in the RFI medium with a high-grain ration led to mitigating methane production by 38% under *in vitro* conditions [31]. It should be noted that this is the first work where a fermented agricultural residue (Lv-carrier) was supplemented to a total mixed ration (Table 1).

The results suggest that there could be a threshold effect of pure Lv on *in vitro* methane production . Likely, this level would be 100 mg/L [31, 40, 43]. Below this threshold, no significant difference seemed to be detected. In contrast, beyond that threshold, the methanogenesis inhibition holds. One could speculate whether this threshold would be related to possible adsorption of Lv onto solids of the RFI medium, thus decreasing Lv availability by the MA [46, 47]. As suggested above, it would be helpful to conduct more research to distinguish between adsorbed and free Lv in the RFI medium.

## *In vivo* experiments

Experiments under *in vivo* conditions related to Lv have mainly focused on the anti-methanogenic effects of the dietary fermented rice straw as an Lv carrier at different concentrations in ruminants (Table 2). Reported results in the open literature are scarce for large ruminants.

**Table 2:** Effect of lovastatin on *in vivo* ruminal methane emissions

Source of Lv	Animal and experimental diet	Technique to estimate rumen CH_4_	Lv, mg/kg LW^b^	Methane production, g CH_4_/Kg DMI^a^		Reference
				Control	Treatment	
Commercial Lv (98% purity, Yick-Vic Chemicals & Pharmaceuticals Ltd., Hong Kong, China)	Ruminant: Sheep Diet: f:c^c^ ratio of (45:52)	Open-circuit respiratory chamber	1.06	25.1	25.9	[48]
Fermented red rice power (Zhejiang Medicines and Health Products, Hangzhou, China)	Ruminant: Bovine Diet: f:c ratio of (15:85)	Open-circuit respiratory chamber	0.92	20	17.1	[49]
Fermented rice	Ruminant: Sheep Diet: Based on rice (50%) and rice hay (50%)	Sulfur hexafluoride (SF6)	2.26	35.2	24.9	[36]
Red yeast rice	Ruminant: Goats Diet: f:c ratio of (70:30)	Open-circuit respiratory chamber	4.34	20	18.3	[50]
Fermented rice straw	Ruminant: Goats Diet f:c ratio of (40:60)	Open-circuit respiratory chamber	4.14	60	35	[35]
Fermented palm kernel cake	Ruminant: Goats Diet: f:c ratio of (73:27)	Open-circuit respiratory chamber	6	24.23	19.23	[37]

^a^Dry matter intake

^b^Live weight

^c^Forage:concentrate ratio

Only one article has been published in the scientific literature with beef cattle as an animal model. Ramírez-Restrepo et al. [49] evaluated a basal diet supplemented with four increasing levels of red yeast rice (RYR) as a source of Lv on dry matter (DM) intake, live weight (LW) gain, and CH_4_ emissions from cattle. The findings of this study suggest a decrease of CH_4_ g/kg DM intake by 14.5% with RYR (40 g/d to give a dose of Lv, 0.92 mg/kg LW) in a diet f:c ratio of 15:85. However, the DM intake decreased by approximately 50% after 6 days of RYR supplementation with higher levels of RYR (110 and 120 g/d), which represented Lv doses of 2.62 and 2.88 mg/kg LW, respectively. The authors also reported ruminant digestive, muscular, and urinary system disorders that disappeared 3 days after the RYR treatment was withdrawn, although the authors ascribed those disorders to unknown metabolites of *Monascus purpureus* during the rice fermentation, not the Lv.

Regarding research on small ruminants, Morgavi et al. [36] evaluated the anti-methanogenic effect of fermented rice as an Lv-carrier to obtain a dose of Lv 2.26 mg/kg LW, with a diet based on fermented rice and hay (ratio, 50:50). This study showed that the production of rumen methane (g/kg DM intake) was decreased by 30% in sheep. This is in complete agreement with the findings of other studies, in which the effects of Lv produced by SSF on ruminal methanogenesis were examined. First, Mohd Azlan et al. [35] reported a decrease in rumen CH_4_ production (− 42%) when rice straw (40%) was replaced by fermented rice straw in the experimental diet of goats (dose of Lv 4.14 mg/kg LW). Candyrine et al. [37] determined that fermented PKC had a lower ruminal methane inhibition (− 20%) when mixed in diets with an f:c ratio of 50:50 and doses of Lv 4 and 6 mg/kg LW in goats.

Wang et al. [50] evaluated the effect of supplementation of RYR with a dose of Lv 4.34 mg/kg LW in a high-forage diet (70%) for goats. Their results suggested a slight decrease in methane production (CH_4_/DM intake, L/kg) of 14%. However, Klevenhusen et al**.** [48] tested the effect of a dose of commercial Lv (98% purity) lower than the previous studies (0.94 mg/kg LW) on the ruminal methane production of a sheep-fed diet containing an f:c ratio of 48:52. Their results showed no significant effects of commercial Lv on rumen methanogenesis (Table 2).

More recently, the effects of PKC as an Lv-carrier on the skeletal muscles of goats were examined [51]. The goats were fed a ration characterized by an f:c ratio (77:23) with three proportions of PKC (fermented with *A. terreus*) to provide 2, 4, and 6 mg Lv/kg LW. This study showed degeneration in goats’ selected muscles at the highest level of Lv. It was concluded that supplementing fermented PKC at Lv dose > 4 mg/kg LW negatively affected the health and welfare of the treated goats. However, the adverse effects of Lv on the muscles of treated goats cannot be attributed to Lv per se because the experiment did not test a positive control with pure Lv.

The experiments using fermented agricultural residues as Lv carriers suggested an anti-methanogenic effect on cattle, sheep, and goats with a range of Lv doses of 0.92 to 6 mg/kg LW (Table 2). Unfortunately, there are no systematic studies that compare the effect of the same dose of Lv on the ruminal methanogenesis in the significant domestic ruminants (cattle, sheep, and goats) simultaneously. Table 2 shows that an Lv 4 mg/kg LW dose was evaluated on goats [35, 37] but not sheep and cattle. In contrast, the dose of 1 mg/kg LW was evaluated on cattle and sheep [48, 49] but not on goats. Thus, no firm trends can be appreciated.

Regarding the influence of feed composition on the Lv effects on ruminal fermentation, as highlighted in Table 1, *in vitro* trials suggest a trend in the anti-methanogenic effect of Lv-dose (> 100 mg/L), which is independent of substrate type either low- or high-concentrate diets. Thus, it seems that there is no effect of the feed composition associated with Lv as a methane inhibitor treatment [31, 40, 43].

On the other hand, *in vivo* trials with dietary fermented rice straw showed that Lv concentration higher than 0.92 (mg/kg LW ) was associated with methanogenesis inhibition [35, 36, 50]; however, no clear trends on a possible effect of feed composition were found.

It could be recommendable to obtain more experimental evidence on the effect of feed composition when Lv was added, both at *in vitro* and *in vivo* studies. The underlying hypothesis would be that feed composition could influence Lv availability, which would have an effect on Lv efficacy in ruminal fermentation.

## Effect of lovastatin on fermentation and rumen digestibility

As far as VFA production is concerned, Wang et al. [50] and Klevenhusen et al. [48] found no significant effects of pure Lv and RYR treatments on the production and profile of VFA in goats and sheep, respectively. It was demonstrated a decrease in acetate molar proportion (*P* < 0.05) and a slight increase in molar proportions of butyrate and propionate when sheep fed fermented rice [36]. Nevertheless, a significant abatement in the butyrate molar proportion was observed when cattle and goats were fed diets with fermented rice straw and RYR as Lv-carriers by Mohd Azlan et al. [35] and Ramírez-Restrepo et al. [49], respectively.

The effect of Lv on *in vitro* the VFA pattern has still not been identified (Table 3), the proportion of propionate was increased. In contrast, acetate and butyrate proportions were reduced by simvastatin and atorvastatin at 100 mg/L; the latter also reduced the total VFA (*P* < 0.05) [43]. However, a similar dose of Lv (150 mg/L) did not affect the production and profile of VFA, although methanogenesis was inhibited (*P* < 0.05) [40].

**Table 3:** Effects of lovastatin on rumen fermentation variables

pH	NH_3_-N	Total VFA	Acetate	Propionate	Butyrate	A:P^a^	Digestibility	Reference
***In vitro*** **experiments**								
≈	≈	≈	≈	≈	≈	≈	≈	[39]
≈	↓	≈	↑	↓	≈	NR^b^	≈	[40]
↑	NR	≈	≈	↓	≈	≈	↑	[34]
≈	NR	≈	≈	≈	≈	NR	≈	[41]
NR	NR	≈	↓	≈	≈	≈	≈	[36]
≈	↑	≈	↓	↑	≈	NR	↑	[42]
≈	NR	↓	↓	↑	≈	↓	NR	[31]
≈	≈	↓	↓	↑	↓	↓	≈	[43]
***In vivo*** **experiments**								
≈	≈	≈	≈	≈	≈	≈	≈	[48]
NR	NR	↓	≈	≈	≈	≈	NR	[49]
NR	NR	↓	↓	≈	≈	↓	NR	[36]
NR	≈	≈	≈	≈	≈	↓	≈	[50]
NR	NR	≈	≈	↑	↑	↑	↑	[35]
≈	NR	≈	≈	↑	≈	↓	≈	[37]

^a^Acetic to propionic acid ratio

^b^*NR*, Not reported; ↓, decreased; ↑, increased; ≈ unchanged

**Fig. 2:**
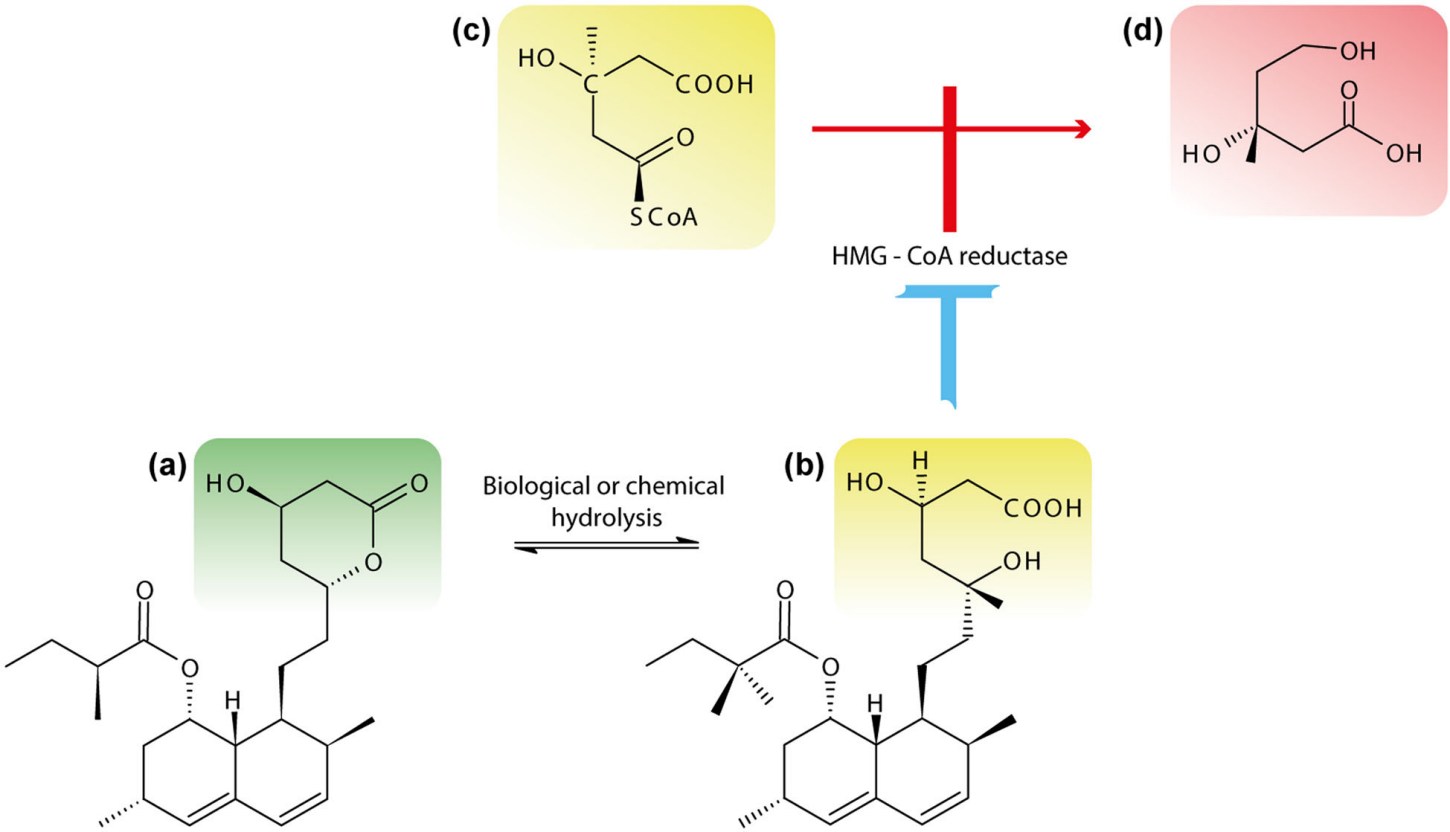
Hydrolysis of lovastatin lactone and the similarity between the chemical structures of lovastatin β-hydroxy acid and the HMG-CoA. **a** Lovastatin lactone, **b** Lovastatin β-hydroxy acid, **c** HMGCoA, and **d** Mevalonate. Adapted from Syed and Ponnisamy [53]

Among the key factors that could influence the anti-methanogenic effect of Lv, we can distinguish (*i*) solubility of Lv and availability, (*ii*) chemical form of Lv, and (*iii*) stability of Lv.

(*i*) According to the biopharmaceutical classification system, statins are class II drugs, poorly water-soluble, and high transport thru biological membranes [52]. These factors are associated with Lv anti-methanogenic effectiveness in the rumen ecosystem. In this context, most (*in vitro* and *in vivo*) trials were conducted with lipophilic statins, for instance, cerivastatin, atorvastatin, rosuvastatin, Lv, and simvastatin [31, 34, 40, 41]. There was limited evidence of the experiments with hydrophilic statins (pravastatin and rosuvastatin) [43]. Lv can easily cross lipid membranes [25] because of its lipophilicity revealed by a poor water solubility of 0.4 mg/L [23, 52]; however, its biological availability could be negatively affected by its poor water solubility.

(*ii*) Another issue that our research group has noted is the dosage form of Lv in ruminal fermentation experiments (Fig. 2): the pure Lv commonly is in lactone form “prodrug” and water-insoluble [54]; there are some ways to activated into its β-hydroxy acid and water-soluble form [55]. However, the studies of ruminal fermentation when pure Lv inhibits methanogenesis did not report Lv chemical activation. Thus, it can be speculated that Lv β-hydroxy could be a better alternative to mitigate rumen methane, at least in short-term trials.

On the other hand, the fungal SSF produced up to 90% of Lv β-hydroxy acid form [53]; as a result, higher availability of active metabolite Lv in the rumen medium is expected. This difference in Lv chemical form appears to explain better results in favor of agricultural residues as Lv-carriers for treating methane mitigation in ruminants.

(*iii*) Another factor is the stability of Lv during the ruminal fermentation trials. Although *in vitro* tests last 3 days or less, possible degradation of Lv and the associated decrease of its concentration could occur in this period. Recent evidence suggests that human gut anaerobes catalyzed the conversion of Lv lactone into Lv β-hydroxy acid [38]. Interestingly, Beltrán et al. [56] demonstrated that Lv incubated in phosphate buffer solution with similar conditions to those of the rumen environment such as temperature 37 °C, pH 7, in the absence of microbiota (abiotic control), resulting in the transformation of Lv lactone into its β-hydroxy acid form. Moreover, the authors demonstrated that experiments of human gut spiked with Lv β-hydroxy acid form could transform the latter to other unknown metabolites.

Unfortunately, stability studies of Lv and its chemical forms in ruminal medium and fermentation conditions are lacking.

In terms of *in vivo* digestibility studies associated with the anti-methanogenic activity of Lv, small sample sizes are a limitation on this issue (Table 3). Moreover, most of these experiments used fermented agricultural residues as Lv-carriers. Wang et al. [50] reported that the DM digestibility was not significantly affected by replacing rice straw (8.2%) with fermented rice straw to obtain a dose of Lv (4.34 mg/kg LW) in an f:c ratio (70:30) diet for goats. This was consistent with results by Candyrine et al. [37], who found that replacing up to 26.3% of PKC by fermented PKC (to obtain a dose of Lv 6 mg/kg LW) in an f:c ratio diet (77:23) did not negatively affect the digestibility of DM. In this context, Mohd Azlan et al. [35] observed that fermented rice straw increased the digestibility of DM by 13% (*P* < 0.05) in goats fed a diet containing 82% of neutral detergent fiber. Also, this experiment showed that the *A. terreus* strain degraded hemicellulose components of the fermented rice straw during the SSF bioprocess. An increase in the digestibility might be due to the production of enzymes cellulases, xylanases, and phenoloxidases by *A. terreus* strains, which would enhance the digestion of structural carbohydrates from lignocellulose feeds [57, 58].

Despite this, *in vitro* and *in vivo* trials showed that pure Lv and simvastatin (100 mg/L culture media and 1.06 mg/kg LW ) did not affect the nutrient digestibility of diets containing 70 and 48% of forage, respectively [43, 48].

In terms of pH and concentration of ammonia nitrogen, no effects were observed from the inclusion of pure Lv or fermented rice to ruminant rations under *in vivo* conditions [48, 50].

## Effect of lovastatin on rumen microbiota

Initial work in rumen microbiology focused primarily on cultivating bacteria for a comprehensive understanding of fermentative metabolism [59, 60]. Afterward, cultured or uncultured microorganisms were subjected to molecular genetic methods to identify and quantify [61]. More recently, the meta-omic integration methodology has provided a more useful characterization of microbiomes (e.g., to identity microbial and predictive metabolomic profile) [62–64].

Within this framework, only three works have evaluated Lv impacts on rumen microbiota using high-throughput sequencing technology [31, 37, 50].

Candyrine et al. [37] assessed the rumen archaea and bacteria composition of goats when fermented PKC inhibited methanogenesis. They found that this did not affect the α (Simpson, Shannon, and Chao 1) and β diversity indices (Principal Coordinate Analysis using Bray-Curtis dissimilarity) concerning the relative abundances of rumen microbiota. Unfortunately, biostatistical analyses were not reported. These results were consistent with those reported by Ábrego-García [31], who observed no significant differences in the α and β diversity indices. However, the authors observed some evidence of differences between selected microbial abundances of the fermented oat straw as an Lv-carrier (150 mg/L) and the control groups. For instance, *Prevotella* abundance was significantly reduced. The abundances of the Ruminococcaceae family and the genus *Ruminococcus* were increased (*P* < 0.05), whereas the Euryarchaeota phylum was reduced by 38%.

The experiment of Wang et al. [50] was limited to the rumen archaea composition. They reported that the Chao 1 alpha diversity was not affected; however, the Shannon-Wiener index was higher in the RYR treatment. Furthermore, the relative abundance of *Methanobrevibacter* was significantly decreased but increased in genus *Methanomicrobium* for the anti-methanogenic treatment. Due to the limited studies on this topic, the information regarding rumen microbial diversity was organized as classic and molecular biology techniques and discussed below.

As expected, *in vivo* and *in vitro* trials demonstrated that populations of archaea and total methanogens, as well as *Methanobacteriales*, were reduced (*P* < 0.05) when methanogenesis also declined due to the Lv treatments [34–36].

### Rumen bacteria

The effectiveness of fermented rice straw and fermented rice supplements mixed with diets for small ruminants (doses of Lv 4.14 and 2.26 mg/kg LW) showed an increase (*P* < 0.05) in the concentration of rumen bacteria of sheep and goats, respectively [35, 36]. These findings were congruent with those of Soliva et al. [40], who used pure Lv (150 mg/L) for *in vitro* ruminal fermentation and demonstrated an increase in the bacteria population. However, Jahromi et al. [34] for *in vitro* experiments reported a significant decrease in the rumen bacteria population with fermented rice straw and extracts of this fermented substrate as treatments for methane mitigation.

### Cellulolytic/fibrinolytic bacteria

No inhibitory effect of pure Lv on the concentration of cellulolytic/fibrinolytic bacteria was observed. This fact was initially established in cultures of *B. fibrisolvens*, *R. albus*, *R. flavefasciens*, *F. succinogenes*, and *S. ruminantium* with a dose of 4 mg/L culture medium [26]. Nevertheless, a possible increase in cellulolytic/fibrinolytic bacteria in the rumen was only associated with fermented agricultural residues as Lv carriers. For instance, Mohd Azlan et al. [35] reported that the *R. albus* population increased (cells/mL) three times over with an Lv dose of 4.14 mg/animal/d in goats. In another study into *in vitro* methane mitigation, it was determined that the concentration of *R. albus* increased (*P* < 0.05), but *F. succinogenes* was reduced with a dose of Lv 4.3 mg/L RFI medium [34].

### Fungi

There is no general agreement about the effect of Lv on rumen fungi. Detailed examination of works by Soliva et al. [40] and Jahromi et al. [34] demonstrated a significant decrease in anaerobic fungi when methanogenesis was significantly inhibited using pure Lv (150 mg/L) and fermented rice straw as an Lv-carrier (4.3 mg/L), respectively. However, a ten-fold increase in the anaerobic fungus population was observed with the fermented rice straw treatment (dose of Lv 4.14 mg/kg LW) compared to the control group in goats [35].

### Protozoa

Mohd Azlan et al. [35] and Morgavi et al. [36] carried out series of experiments to mitigate methane production with fermented rice and rice straw treatments produced by SSF with *M. porpureus spp* and *A. terreus* (doses of Lv 2.26 and 4.14 mg/kg LW) in sheep and goats, respectively. No statistically significant differences in the rumen protozoa population were found between the treatment and control groups in those studies. This result was also observed for *in vitro* trials with RFI from sheep. Fermented rice straw or ethanolic extracts of fermented rice straw were evaluated on the population of protozoa [34]. Finally, there were no reports in the open literature on the effects on the rumen protozoa population when methane inhibition was significantly reduced by pure Lv treatment.

## Conclusion

Lv treatment had a strong anti-methanogenic effect on pure strains of MA. However, there are uncertainties about results for *in vitro* fermentation with complex substrates and RFI.

On the other hand, the SSF of agricultural residues is a bioprocess that can be adopted for Lv production at a low cost. Those fermented agricultural as Lv-carriers fed to ruminants demonstrated a reliable decrease in ruminal methane emission. However, a remarkable feature of the currently available literature is the lack of positive control (pure Lv) in animal experiments.

The experimental evidence for *in vitro* trials showed that the VFA production was not affected by Lv; however, the results from *in vivo* trials demonstrated that production of VFA was decreased. The *in vitro* and *in vivo* DM digestibility was not negatively affected by Lv.

Regarding rumen microbiota, no differences were detected in alpha and beta diversity associated with Lv treatment, but it induced some changes in the relative abundance. Lv did not have an inhibitory effect on ruminal eubacteria but, there is insufficient evidence to determine its relationship to fungi and ruminal protozoa.

Additionally, further research is needed on the following issues: (*i*) Lv biodegradation products and stability, as well as its adsorption onto the solid matter in the rumen, should be assessed to determine the “available” or effective Lv concentration, and (*ii*) to assess whether the effect of Lv on ruminal fermentation also depends on the feed composition and different ruminants.

## Data Availability

None.

## Abbreviations

DM: Dry matter
DMD: Dry matter digestibility
f:c ratio: Fiber to concentrate ratio
GHG: Greenhouse gas
HMG-CoA: 3-Hydroxy-3-methylglutaryl CoA
Lv: Lovastatin
LW: Live weight
MA: Methanogenic archaea
PKC: Palm kernel cake
RFI: Rumen fluid inoculum
RYR: Red yeast rice
SSF: Solid-state fermentation
VFA: Volatile fatty acids

## References

[1] Ripple WJ, Smith P, Haberl H, Montzka SA, McAlpine C, Boucher DH. Ruminants, climate change and climate policy. Nat Clim Chang. 2014;4(1):2–5. 10.1038/nclimate2081.

[2] Herrero M, Henderson B, Havlík P, Thornton PK, Conant RT, Smith P, Wirsenius S, Hristov AN, Gerber P, Gill M, Butterbach-Bahl K, Valin H, Garnett T, Stehfest E (2016). Greenhouse gas mitigation potentials in the livestock sector. Nat Clim Chang.

[3] Victor DG, Edenhofer O (2014). Introductory chapter. Climate change 2014: mitigation of climate change. Contribution of working group III to the fifth assessment report of the intergovernmental panel on climate change: 2014.

[4] Saunois M, Stavert AR, Poulter B, Bousquet P, Canadell JG, Jackson RB, Raymond PA, Dlugokencky EJ, Houweling S, Patra PK, Ciais P, Arora VK, Bastviken D, Bergamaschi P, Blake DR, Brailsford G, Bruhwiler L, Carlson KM, Carrol M, Castaldi S, Chandra N, Crevoisier C, Crill PM, Covey K, Curry CL, Etiope G, Frankenberg C, Gedney N, Hegglin MI, Höglund-Isaksson L, Hugelius G, Ishizawa M, Ito A, Janssens-Maenhout G, Jensen KM, Joos F, Kleinen T, Krummel PB, Langenfelds RL, Laruelle GG, Liu L, Machida T, Maksyutov S, McDonald KC, McNorton J, Miller PA, Melton JR, Morino I, Müller J, Murguia-Flores F, Naik V, Niwa Y, Noce S, O'Doherty S, Parker RJ, Peng C, Peng S, Peters GP, Prigent C, Prinn R, Ramonet M, Regnier P, Riley WJ, Rosentreter JA, Segers A, Simpson IJ, Shi H, Smith SJ, Steele LP, Thornton BF, Tian H, Tohjima Y, Tubiello FN, Tsuruta A, Viovy N, Voulgarakis A, Weber TS, van Weele M, van der Werf GR, Weiss RF, Worthy D, Wunch D, Yin Y, Yoshida Y, Zhang W, Zhang Z, Zhao Y, Zheng B, Zhu Q, Zhu Q, Zhuang Q (2020). The global methane budget 2000-2017. Earth Syst Sci Data.

[5] Gerber PJ, Steinfeld H, Henderson B, Mottet A, Opio C, Dijkman J, Falcucci A, Tempio G (2013). Implications for policy-marking. Tackling Climate Change through Livestock-A global assessment of emissions and mitigation opportunities.

[6] Owens FN, Basalan M, Millen D, De Beni Arrigoni M, Lauritani Pacheco RD (2016). Ruminal fermentation. Rumenology.

[7] Tapio I, Snelling TJ, Strozzi FWR. The ruminal microbiome associated with methane emissions from ruminant livestock. J Anim Sci Biotechnol. 2017;8:7. 10.1186/s40104-017-0141-0. 10.1186/s40104-017-0141-0 PMC5244708 28123698

[8] Johnson KA, Jhonson DE (1995). Methane emissions from cattle. J Anim Sci.

[9] Patra A, Park T, Kim M, Yu Z. Rumen methanogens and mitigation of methane emission by anti-methanogenic compounds and substances. J Anim Sci Biotechnol. 2017;8:13. 10.1186/s40104-017-0145-9. 10.1186/s40104-017-0145-9 PMC5270371 28149512

[10] Beauchemin KA, Ungerfeld EM, Eckard RJ, Wang M. Review: fifty years of research on rumen methanogenesis: lessons learned and future challenges for mitigation. Animal. 2020;14 Suppl 1:S2–S16. 10.1017/S1751731119003100. 10.1017/S1751731119003100 32024560

[11] Mahdavi A, Bagherniya M, Fakheran O, Reiner Ž, Xu S, Sahebkar A (2020). Medicinal plants and bioactive natural compounds as inhibitors of HMG-CoA reductase: a literature review. BioFactors.

[12] Jain S, Caforio A, Driessen AJ (2014). Biosynthesis of archaeal membrane ether lipids. Front Microbiol.

[13] Goopy JP (2019). Creating a low enteric methane emission ruminant: what is the evidence of success to the present and prospects for developing economies?. Anim Prod Sci.

[14] Haque MN. Dietary manipulation: a sustainable way to mitigate methane emissions from ruminants. J Anim Sci Technol. 2018;60:15. 10.1186/s40781-018-0175-7. 10.1186/s40781-018-0175-7 PMC6004689 29946475

[15] Konings WN, Albers SV, Koning S, Driessen AJ (2002). The cell membrane plays a crucial role in survival of bacteria and archaea in extreme environments. Antonie Van Leeuwenhoek.

[16] Sûstar V, Zelko J, Lopalco P, Lobasso S, Ota AUN (2012). Morphology, biophysical properties and protein mediated fusion of Archaeosomes. PLoS One.

[17] Vickers CE, Sabri S (2015). Isoprene. Adv Biochem Engin/Biotechnol.

[18] Knappy CS, Nunn CE, Morgan HW, Keely BJ. The major lipid cores of the archaeon Ignisphaera aggregans: implications for the phylogeny and biosynthesis of glycerol monoalkyl glycerol tetraether isoprenoid lipids. Extremophiles. 2011;15(4):517–28. 10.1007/s00792-011-0382-3. 10.1007/s00792-011-0382-3 21630026

[19] Goswami S, Vidyarthi AS, Bhunia BMT (2012). A review on lovastatin and its production. J Biochem Tech.

[20] Subhan M, Faryal R, Macreadie I (2016). Exploitation of Aspergillus terreus for the production of natural statins. J Fungi.

[21] Salvador-Castell M, Tourte M, Oger PM (2019). In search for the membrane regulators of archaea. Int J Mol Sci.

[22] Wu Y, Xiong F, Chen F (2015). Stereoselective synthesis of 3-hydroxy-3- methylglutaryl– coenzyme a reductase inhibitors. Tetrahedron.

[23] Ying J, Du LD, Du GH, Du G-H (2018). Lovastatin. Natural small molecule drugs from plants.

[24] Matsumi R, Atomi H, Driessen AJM, van der Oost J (2011). Isoprenoid biosynthesis in Archaea – biochemical and evolutionary implications. Res Microbiol.

[25] Gottlieb K, Wacher V, Sliman J, Pimentel M (2016). Review article: inhibition of methanogenic archaea by statins as a targeted management strategy for constipation and related disorders. Aliment Pharmacol Ther.

[26] Miller TL, Wolin MJ (2001). Inhibition of growth of methane-producing bacteria of the ruminant forestomach by hydroxymethylglutaryl-SCoA reductase inhibitors. J Dairy Sci.

[27] Jahromi MF, Liang J, Ho YW, Mohamad R, Goh YM, Shokryazdan P, et al. Lovastatin in Aspergillus terreus: fermented rice straw extracts interferes with methane production and gene expression in Methanobrevibacter smithii. Biomed Res Int. 2013;2013:604721. 10.1155/2013/604721. 10.1155/2013/604721 PMC3655455 23710454

[28] Sharma A, Chaudhary P, Sirohi S, Saxena J (2011). Structure modeling and inhibitor prediction of NADP oxidoreductase enzyme from Methanobrevibacter smithii. Bioinformation.

[29] Muskal SM, Sliman J, Kokai-Kun J, Pimentel M, Wacher V, Gottlieb K (2016). Lovastatin lactone may improve irritable bowel syndrome with constipation (IBS-C) by inhibiting enzymes in the archaeal methanogenesis pathway. F1000Res.

[30] Mulder KC, Mulinari F, Franco OL, Soares MS, Magalhães BS, Parachin NS (2015). Lovastatin production: from molecular basis to industrial process optimization. Biotechnol Adv.

[31] Ábrego-García A, Poggi-Varaldo HM, Mendoza-Vargas A, Mercado-Valle FGR-LE, Ponce-Noyola T, Calva-Calva G. Effects of fermented oat straw as a lovastatin-carrier on in vitro methane production and rumen microbiota. Front Energy Res. 2021;9:630701. 10.3389/fenrg.2021.630701.

[32] Godoy MG, Amorim GM, Barreto MS, Freire DMG, Pandey A, Larroche C, Soccol C (2018). Chapter 10: Agricultural residues as animal feed: Protein enrichment and detoxification using solid-state fermentation. Current Developments in Biotechnology and Bioengineering.

[33] Varadyova Z, Certik M, Jalc D (2018). The possible application of fungal enriched substrates in ruminant nutrition. A review. J Anim Feed Sci.

[34] Jahromi MF, Liang JB, Mohamad R, Goh YM, Shokryazdan P, Ho YW (2013). Lovastatin-enriched rice straw enhances biomass quality and suppresses ruminal methanogenesis. Biomed Res Int.

[35] Mohd Azlan P, Jahromi MF, Ariff MO, Ebrahimi M, Candyrine SCL, Liang JB (2018). Aspergillus terreus treated rice straw suppresses methane production and enhances feed digestibility in goats. Trop Anim Health Prod.

[36] Morgavi DP, Martin C, Boudra H (2013). Fungal secondary metabolites from *Monascus* spp. reduce rumen methane production in vitro and in vivo. J Anim Sci.

[37] Candyrine SCL, Mahadzir MF, Garba S, Jahromi MF, Ebrahimi M, Goh YM, Samsudin AA, Sazili AQ, Chen WL, Ganesh S, Ronimus R, Muetzel S, Liang JB (2018). Effects of naturally-produced lovastatin on feed digestibility, rumen fermentation, microbiota and methane emissions in goats over a 12-week treatment period. PLoS One.

[38] Demonfort Nkamga V, Armstrong N, Drancourt M (2017). In vitro susceptibility of cultured human methanogens to lovastatin. Int J Antimicrob Agents.

[39] Busquet M, Calsamiglia S, Ferret A, Carro MD, Kamel C (2005). Effect of garlic oil and four of its compounds on rumen microbial fermentation. J Dairy Sci.

[40] Soliva CR, Amelchanka SL, Duval SM, Kreuzer M (2011). Ruminal methane inhibition potential of various pure compounds in comparison with garlic oil as determined with a rumen simulation technique (Rusitec). Br J Nutr.

[41] O’Brien M, Navarro-Villa A, Purcell PJ, Boland T, O’kiely P (2014). Reducing in vitro rumen methanogenesis for two contrasting diets using a series of inclusion rates of different additives. Anim Prod Sci.

[42] Khonkhaeng B, Cherdthong A (2020). Improving nutritive value of purple field corn residue and Rice straw by culturing with white-rot Fungi. J Fungi.

[43] Joch M, Vadroňová M, Výborná A, Jochová K. Inhibition of in vitro rumen methane production by three statins. Ann Anim Sci. 2021. 10.2478/aoas-2021-0022.

[44] Monson PA (2012). Understanding adsorption/desorption hysteresis for fluids in mesoporous materials using simple molecular models and classical density functional theory. Micropor Mesopor Mat.

[45] Sathya V, Sinduja M, Kalpana P, Maheswari M, Ramasubramaniyan MR, Mahimairaja S. Strategic study of adsorption and desorption of chromium on vertisols and its implication in developing an effective remediation technology. Int J Environ Anal Chem. 2021. 10.1080/03067319.2021.1940985.

[46] Poggi-Varaldo HM, Rinderknecht-Seijas N, Caffarel-Méndez S (2002). Irreversibilidad en el comportamiento adsortivo-desortivo de contaminantes en suelos y sedimentos: evaluación cuantitativa por medio de un coeficiente de histéresis diferencial. Interciencia.

[47] Robles-González IR, Ríos-Leal E, Galíndez-Mayer JM, Caffarel-Méndez S, Barrera-Cortés J, Esparza-García F, Poggi-Varaldo HM (2006). Adsorptive-desorptive behaviour of lindane in an agricultural soil. Interciencia.

[48] Klevenhusen F, Duval S, Zeitz JO, Kreuzer M, Soliva CR (2011). Diallyl disulphide and lovastatin: effects on energy and protein utilisation in, as well as methane emission from, sheep. Arch Anim Nutr.

[49] Ramírez-Restrepo CA, O’neill CJ, López-Villalobos N, Padmanabha J, Mcsweeney C (2014). Tropical cattle methane emissions: the role of natural statins supplementation. Anim Prod Sci.

[50] Wang LZ, Zhou ML, Wang JW, Wu D, Yan T (2016). The effect of dietary replacement of ordinary rice with red yeast rice on nutrient utilization, enteric methane emission and rumen archaeal diversity in goats. PLoS One.

[51] Leo TK, Garba S, Abubakar D, Sazili AQ, Candyrine SCL (2020). Naturally produced lovastatin modifies the histology and proteome profile of goat skeletal muscle. Animals.

[52] Kajdič S, Zupančič S, Roškar R, Kocbek P (2019). The potential of nanofibers to increase solubility and dissolution rate of the poorly soluble and chemically unstable drug lovastatin. Int J Pharm.

[53] Syed MB, Ponnusamy T (2018). Bioconversion of mevastatin to pravastatin by various microorganisms and its applications–a review. Biocatal Agric Biotechnol.

[54] Zhang Y, Zhang H, Che E, Zhang L, Han J, Yang Y, Wang S, Zhang M, Gao C (2015). Development of novel mesoporous nanomatrix-supported lipid bilayers for oral sustained delivery of the water-insoluble drug, lovastatin. Colloids Surf B Biointerfaces.

[55] Qiao L, Xie D, Liu Q, Zou J, Shen Z, Dai J (2012). Microbial transformation of lovastatin by Beauveria bassiana. Acta Pharm Sin B.

[56] Beltrán D, Frutos-Lisón MD, Espín JC, García-Villalba R (2019). Re-examining the role of the gut microbiota in the conversion of the lipid-lowering statin monacolin K (lovastatin) into its active β-hydroxy acid metabolite. Food Funct.

[57] Abrão FO, Duarte ER, Pessoa MS, Dos Santos VL, De Freitas LF, Barros KDO (2017). Notable fibrolytic enzyme production by Aspergillus spp. isolates from the gastrointestinal tract of beef cattle fed in lignified pastures. PLoS One.

[58] Londoño-Hernandez L, Ruiz HA, Toro CR, Ascacio-Valdes A, Rodriguez-Herrera R, Aguilera-Carbo A, Tubio G, Pico G, Prado-Barragan A, Gutierrez-Sanchez G, Aguilar CN, Arora N, Mishra J, Mishra V (2020). Advantages and Progress innovations of solid-state fermentation to produce industrial enzymes. Microbial enzymes: roles and applications in industries. Microorganisms for sustainability.

[59] Hungate RE, Bryant M, Mah RA (1964). The rumen Bacteria and Protozoa. Annu Rev Microbiol.

[60] Hobson PN. Rumen Bacteria. Methods Microbiol. 1969:133–49. 10.1016/S0580-9517(08)70504-X.

[61] Knight IT, Colwell RR, Grimes D (2000). Molecular genetic methods for detection and identification of viable but Nonculturable. Microorganisms Nonculturable microorganisms in the environment.

[62] Muller EEL, Faust K, Widder S, Herold M, Martínez Arbas S, Wilmes P (2018). Using metabolic networks to resolve ecological properties of microbiomes. Curr Opin Syst Biol.

[63] Gharechahi J, Vahidi MF, Bahram M, Han JL, Ding XZ, Salekdeh GH (2021). Metagenomic analysis reveals a dynamic microbiome with diversified adaptive functions to utilize high lignocellulosic forages in the cattle rumen. ISME J.

[64] He B, Jin S, Cao J, Mi L, Wang J (2019). Metatranscriptomics of the Hu sheep rumen microbiome reveals novel cellulases. Biotechnol Biofuels.

